# Risk Factors for Carbetocin Failure after a Cesarean Section: Is Obesity One of Them?

**DOI:** 10.3390/jcm10173767

**Published:** 2021-08-24

**Authors:** Manon Degez, Lucie Planche, Agnès Dorion, Alexis Duchalais, Emelyne Lefizelier, Guillaume Ducarme

**Affiliations:** 1 Department of Obstetrics and Gynecology, Centre Hospitalier Departemental, 85000 La Roche sur Yon, France; manondegez@msn.com (M.D.); emelyne.lefizelier@ght85.fr (E.L.); 2 Clinical Research Center, Centre Hospitalier Departemental, 85000 La Roche sur Yon, France; lucie.planche@ght85.fr (L.P.); agnes.dorion@ght85.fr (A.D.); 3 Department of Anesthesiology, Centre Hospitalier Departemental, 85000 La Roche sur Yon, France; alexis.duchalais@ght85.fr

**Keywords:** carbetocin, failure, obesity, postpartum hemorrhage, prevention

## Abstract

Obese pregnant women have increased rates of fetal macrosomia, long labor, and cesarean sections, which lead to an increased risk of postpartum hemorrhage (PPH). Carbetocin is useful for the prevention of PPH after a cesarean section. Our study aimed to investigate predictors of carbetocin failure after a cesarean section, and specifically whether obesity is associated with carbetocin failure. We retrospectively analyzed all women who received carbetocin after a cesarean section. Carbetocin failure was defined as changes in hematocrit and hemoglobin, blood loss ≥ 1000 mL, and the need for an additional uterotonic agent or second-line therapies for persistent PPH. Univariate and multivariate analyses were performed to investigate predictors of carbetocin failure. The study included 600 women, with 131 (21.8%) obese women. Overall, 44 (7.3%) carbetocin failures were reported, and rates of obese women were similar between groups (carbetocin failure, 11.4% vs. 22.9%; *p* = 0.08). Previous PPH (*p* < 0.001), a cesarean section during labor (*p* = 0.01), cervical ripening (*p* = 0.02), and birthweight (*p* = 0.01) were significantly different between groups. In the multivariable logistic regression analysis adjusted for potential confounders, cervical ripening (adjusted odds ratio (OR) 2.23, 95% confidence interval (CI) 1.01–4.80), compared with spontaneous labor, was significantly associated with carbetocin failure. Obesity was not associated with carbetocin failure after cesarean sections.

## 1. Introduction

The prevalence of obese women (body mass index, BMI ≥ 30 kg/m²) in childbearing age is increasing in all western societies [1]. A rise in the cesarean section rate was found in all series, with a 1 kg/m² increase in BMI leading to a 7% increase in the risk of cesarean sections [2,3]. These women seem to have an increased rate of fetal macrosomia, preeclampsia, induced labor, long labor, and cesarean sections [4,5], and then an increased risk of postpartum hemorrhage (PPH) [6]. Moreover, Doherty et al. [7] found a significant association between obesity and increased operating time, which was associated with increased blood loss.

Carbetocin is a synthetic human oxytocin analogue with a longer duration of action than oxytocin (approximately 5 h instead of 90 min). The onset of uterine contractions following the administration of carbetocin is fast and firm contractions are obtained within 2 min. Carbetocin is currently indicated for the prevention of uterine atony after delivery by cesarean section in spinal or epidural anesthesia. Systematic reviews demonstrated that carbetocin was more effective and safer than oxytocin for reducing the need for additional uterotonic drugs and uterine massage after cesarean delivery [8,9,10,11]. In the case of carbetocin inefficiency, a management policy for persistent PPH during a cesarean section was applied with an additional uterotonic agent (sulprostone) and second-line therapies (Bakri balloon, uterine compression sutures, uterine artery embolization, and peripartum hysterectomy) after the failure of uterine massage and uterotonic agents to stop bleeding [12]. However, it is unclear whether carbetocin is more effective than oxytocin for the prevention of PPH in obese women. To our knowledge, only one double-blinded randomized-controlled trial has compared the efficacy and safety of a single intravenous (IV) bolus dose of carbetocin versus IV oxytocin infusion in the prevention of PPH among 180 obese nulliparous women undergoing emergency cesarean delivery [13]. More severe PPH ≥ 1000 mL, and more transfusions in the oxytocin group were observed (*p* = 0.03 and *p* = 0.04, respectively). None from the carbetocin group versus 71.5% in the oxytocin group needed additional uterotonics (*p* < 0.01) [13]. In our hospital, firstly, carbetocin was introduced in May 2017 in the prevention of PPH after planned cesarean sections, and since January 2018, all women have received carbetocin during cesarean sections regardless of their indications. The aim of our study was to investigate predictive factors of carbetocin failure after cesarean sections and, specifically, whether obesity is associated with carbetocin failure.

## 2. Methods

### 2.1. Patient Selection

We conducted a retrospective observational study from 1 May 2017 to 31 December 2019. The study group included all women with a live singleton fetus, who gave birth by cesarean section with carbetocin administration, regardless of the indication of the cesarean section and the gestational age at delivery, from 1 May 2017 to 31 December 2019 at one tertiary care hospital.

We excluded women without carbetocin administration during cesarean sections, without gestational age dating (crown-to-rump length at a first-trimester ultrasound examination or fetal biometry before 24 weeks), and women with medical-indicated second trimester termination of pregnancy, intra uterine death or fetal loss before 22 weeks.

The recruitment was carried out retrospectively from existing patient records (birth book, medical record software, and paper medical records). During cesarean sections, carbetocin was administered as an IV single dose (100 µg) after cord clamping, and the inefficiency of carbetocin with persistent PPH was managed, as usual, with an additional uterotonic agent (sulprostone) and second-line therapies (Bakri balloon, uterine compression sutures, uterine artery embolization, and peripartum hysterectomy) for management of massive persistent PPH after the failure of uterine massage and uterotonic agents to stop bleeding.

This present study was conducted in accordance with the French approved guidelines. All participants received written information about the study using institutional review board-approved documents. Written consent is not required for a retrospective study according to French law, but each woman obtained the opportunity to opt out of the analysis. The study protocol was approved by a Research Ethics Committee (Groupe Nantais d’Ethique dans le Domaine de la Santé (GNED)) on January 2020 before the beginning of the study (n° 2020-A2201).

### 2.2. Data Collection

Maternal sociodemographic characteristics, information regarding pregnancy follow-up, and standard perinatal outcomes were collected retrospectively by one obstetrician (M.D.) from the electronic medical record database of the hospital. Maternal characteristics collected included maternal prepregnancy age, BMI (calculated as weight (kg)/(height (m))²), based on height and the first weight noted in the obstetric record), geographic origin (North Africa, Sub-Saharan Africa, Hispanic, Asian, Overseas departments, Caucasian), parity, known uterine malformation, and previous uterine scars (previous cesarean section or myomectomy).

Pregnancy characteristics collected included type of pregnancy (singleton or twin), gestational diabetes mellitus (GDM) [14], pregnancy-associated hypertensive disorders (determined by hypertension without proteinuria or preeclampsia (hypertension and proteinuria after 20 weeks’ gestation in a previously normotensive woman)) [15], gestational weight gain (GWG), intrahepatic cholestasis of pregnancy (ICP, characterized by pruritus with onset in the second or third trimester of pregnancy and elevated serum aminotransferases and bile acid levels), placenta localization at the third trimester (praevia or not), antenatal suspicion of macrosomia at the third trimester (defined as an ultrasonographic estimated fetal weight > 90th centile adjusting for gestational age and sex [16]), polyhydramnios at the third trimester, pre-delivery hemoglobin (anemia defined by hemoglobin <11 g/dL before delivery [17]), and pre-delivery hematocrit. Intrapartum variables collected included gestational age at delivery (determined by the craniocaudal length at a first-trimester ultrasound examination), type of labor (spontaneous or induced, planned cesarean delivery), mode of delivery (spontaneous or operative vaginal delivery, or cesarean section during labor), indication of cesarean delivery, type of anesthesia, chorioamnionitis, total estimated blood loss, PPH (defined as bleeding 500 mL or higher), need for additional uterotonics (sulprostone), second-line therapies (Bakri balloon, uterine compression sutures, uterine artery embolization, and peripartum hysterectomy), and birth weight. In addition, maternal characteristics collected in postpartum period included length of stay, need for iron supplementation (oral or IV), post-delivery (day-1) hemoglobin and hematocrit, blood transfusion, and intensive care unit (ICU) admission.

Our primary objective was to investigate predictive factors of carbetocin failure after cesarean section and, specifically, whether obesity is associated with carbetocin failure. The primary endpoint was a composite criteria for carbetocin failure defined by at least one of the following criteria: change in hematocrit pre- and post-delivery ≥10%, change in hemoglobin pre- and post-delivery ≥4 g/dL, estimated total blood loss ≥1000 mL, need for an additional uterotonic agent (sulprostone), and need for second-line therapies (Bakri balloon, uterine compression sutures, uterine artery embolization, and peripartum hysterectomy) for management of persistent PPH.

The secondary objectives were to investigate the indirect signs of PPH and its consequences after cesarean section with carbetocin administration. The secondary endpoints were the need for iron supplementation (oral or IV) in postpartum period due to anemia, prolongation of hospital stay due to PPH and/or anemia, and the need of blood transfusion.

The cesarean section rate was about 14.5–15% out of 2500–2600 deliveries each year in our tertiary hospital, that represented about 380–390 cesareans deliveries per year. The study planned to include approximately 600 women who received carbetocin between May 2017 and December 2019.

### 2.3. Statistical Analysis

Continuous data were described by their means ± standard deviations and compared by t tests (or Mann–Whitney tests when appropriate), and categorical data were described by percentages and compared by χ² tests (or Fisher’s exact tests when appropriate). Maternal and perinatal outcomes were compared according to carbetocin efficacy. Risk factors associated with carbetocin failure were analyzed using univariate logistic regressions. A multivariate logistic regression model was then constructed from the univariate significant variables. All analyses were carried out using R software (version 4.0.5; The R Foundation for Statistical Computing, Vienna, Austria, 31 March 2020; The R Foundation for Statistical Computing, developed at Bell Laboratories (Lucent Technologies) by John Chambers and colleagues). *p* values < 0.05 were considered to be statistically significant.

## 3. Results

During the study period, 6922 live births occurred in our hospital, 987 women (14.3%) were delivered by cesarean section, and 600 women (60.8%) received carbetocin during the cesarean section (265 women from May 2017 to January 2018, and 335 women from January 2018 to December 2019). Among all included women with carbetocin administration, only 44 (7.3%) had carbetocin failure, and 15 of them (34%) did not present estimated total blood loss ≥1000 mL after cesarean delivery.

Table 1 details the maternal and pregnancy characteristics according to the carbetocin efficacy. Sociodemographic characteristics of women with carbetocin failure only differed according to the previous PPH (Table 1).

Table 2 details the labor characteristics and maternal and neonatal outcomes according to the carbetocin efficacy. There were no maternal or perinatal deaths. Women with carbetocin failure differed according to induced labor, cervical ripening, cesarean section during labor, cervical dilatation at the cesarean delivery, and birth weight (Table 2). For cervical ripening, the rate of carbetocin failure was 29.5% (*n* = 13) and did not differ significantly among methods (cervical ripening balloon or vaginal dinoprostone insert). Carbetocin failure was less frequent in the planned cesarean sections (*p* = 0.01).

Among all included women with carbetocin administration, 44 (7.3%) women had carbetocin failure with a mean blood loss of 1232 ± 1004 mL. Sulprostone was required in 20 women after carbetocin failure. Different second-line therapies were required for the management of persistent PPH after failure of uterine massage and sulprostone administration to stop bleeding: Bakri balloon (*n* = 2), uterine compression sutures (*n* = 2), artery ligations (*n* = 3), and peripartum hysterectomy after failure of conservative surgical methods (*n* = 2). Maternal transfusion (11.4% compared to 0.2%), change in hemoglobin (Δ = 3.0 ± 1.2 compared to 1.4 ± 1.0 g/dL), and change in hematocrit (Δ = 8.5 ± 3.5 compared to 3.6 ± 3.0 %) were significantly more frequent in carbetocin failure compared with success (all *p* < 0.001) (Table 2).

In the multivariable logistic regression analysis adjusted for potential confounders, only cervical ripening (adjusted odds ratio (aOR) 2.23, 95% confidence interval (CI) 1.01–4.80), compared with spontaneous labor, was significantly associated with carbetocin failure (Table 3).

## 4. Discussion

In our study, obesity was not associated with a higher rate of carbetocin failure after a cesarean section. Previous PPH, cesarean section during labor, cervical ripening and birthweight were significantly more different between groups. In the multivariable logistic regression analysis adjusted for potential confounders, only cervical ripening (aOR 2.23, 95%CI 1.01–4.80), compared with spontaneous labor, was significantly associated with carbetocin failure after cesarean delivery.

Although the retrospective and monocentric natures of our study make it difficult to extrapolate and compare the results with the literature, it allows us to evaluate the effectiveness of carbetocin in obese women, compared to non-obese women. Nevertheless, we did not find an association between obesity and carbetocin failure. Our results are discordant with the literature. Maternal obesity is associated with an increased adipose tissue and higher serum leptin levels in these women that causes inhibition of uterine contractions (tocolytic effect due to less Ca²^+^ flux in the myometrium) [18,19]. The antagonism between leptin and oxytocin has led to the suggestion that obese women should have longer a birth duration and higher oxytocin need during labor [18,20,21,22]. Obese women seem also to have increased rates of fetal macrosomia, preeclampsia, and GDM, resulting in higher rates of induction of labor and cesarean deliveries [2,3,4,5]. Moreover, in a prospective, consecutive cohort included all 31,341 women giving birth in Wales in 2017, Bell et al. [23] demonstrated that the incidence of PPH varied with the mode of delivery and was more than twice as high for an emergency cesarean section as for an elective cesarean section (19.8% vs. 8.5%). For all of these reasons, obese women seem to have an increased rate of PPH [6,24].

Concerning the comparison of carbetocin vs. oxytocin for prevention of PPH after cesarean delivery, Heslehurst et al. [25] found an increased risk of PPH rising with maternal weight (BMI ≥ 30 kg/m²: OR 1.20 (1.16–1.24) and BMI ≥ 40 kg/m²: OR 1.43 (1.33–1.54). Can this difference be attributed to a lack of potency or an advantage of carbetocin over oxytocin in obesity? El Behery et al. [13] published a study comparing the efficacy of oxytocin and carbetocin in a population of obese women undergoing an emergency cesarean section. More PPH ≥ 1000 mL and more transfusions were observed in the oxytocin group (*p* = 0.03 and *p* = 0.04, respectively). This is the only study in our knowledge comparing the two uterotonics in this specific population. It would seem relevant to us to carry out other comparative studies between the use of carbetocin and oxytocin in obese women in order to determine whether there is a difference in the efficacy of the two uterotonics according to the BMI.

We have also highlighted that PPH was underdiagnosed in our study, with too imprecise evaluation of total blood loss after cesarean delivery in current practice. In our study, 34% of the women who presented a PPH that met our composite clinical and bio-logical criteria were reported with blood loss <1000 mL. Using our composite criteria, we were able to identify women who were not diagnosed with severe PPH after cesarean delivery. Our results are similar to those of others [26,27,28]. Among 106 maternity units in France, Dupont et al. found that one in five severe PPHs were exclusively biologically discovered in the postpartum period [26]. More recently, a secondary analysis of a multicenter randomized controlled trial conducted in five French university hospitals found that undiagnosed abnormal postpartum blood loss occurred in about 1 in 10 vaginal deliveries [28]. Combining clinical and biological parameters seems essential to better identify women who presented PPH after cesarean delivery.

The principal strength of our study is the analysis of predictive carbetocin failure factors after cesarean delivery. To our knowledge, no study has already reported predictors of carbetocin failure. Our study is also a pioneering one because obesity, although regularly described as a risk factor for PPH and obstetric complications [25,29,30,31,32,33,34,35,36], is not well targeted in the literature concerning the prevention of PPH after a cesarean delivery [6,37,38]. A recent double-blind, dose-finding study of carbetocin in women with a BMI ≥ 40 kg/m² undergoing an elective cesarean section demonstrated that the 90% effective dose (ED90) for carbetocin in obese women at an elective cesarean section was approximately four times higher than the previously demonstrated ED90 in non-obese women [37]. In order to elucidate the difference of efficacy of oxytocin vs. carbetocin administration in obese women after cesarean delivery, a prospective randomized controlled trial would be ideal.

Our results must be interpreted in light of certain limitations. First, our study reflects the experience of one tertiary hospital along a three-year period with a large enrolment, but the heterogeneity of carbetocin use over the years makes it difficult to interpret our data accurately. From May 2017 to December 2019, 600 women (60.8%) out of 987 cesarean sections received carbetocin. To explain this, it should be noted that for the period from May to December 2017 (265 cesarean sections), carbetocin was only administered to planned cesarean sections. Since January 2018, it was decided to use carbetocin for all caesarean sections within the limits of its contraindications. In addition, some practitioners tended to use oxytocin more easily, particularly in emergency cesarean sections, out of habit. Then, the number of included women (*n* = 600) with a limited number of carbetocin failure (*n* = 44) in our sample might not have been high enough to reveal a clinically meaningful carbetocin failure after cesarean delivery using a composite (clinical and biological) criteria during the study period and to reveal a statistically effect of maternal and obstetric characteristics that could represent an increased risk factor of carbetocin failure.

## 5. Conclusions

Obesity was not associated with carbetocin failure after cesarean sections. Our results are promising as to the effectiveness of carbetocin in cesarean section in obese women. Other randomized studies between oxytocin and carbetocin would be desirable to confirm the interest in this high-risk population. There is a real public health challenge to adapt our management towards obese women in order to decrease morbidity, particularly with regard to prevention of PPH. In our study, cervical ripening seems to be associated with carbetocin failure. Physicians should be aware about PPH prevention during cesarean sections in these cases. Finally, we have highlighted that PPH after cesarean delivery is still underdiagnosed nowadays with too imprecise an evaluation of blood loss in current practice. Combining clinical and biological parameters seem essential to better identify women who have presented PPH after a cesarean delivery.

## Figures and Tables

**Table 1:** Maternal and pregnancy characteristics according to the carbetocin efficacy.

	No Failure	Failure	*p*-Value
	(*n* = 556)	(*n* = 44)	
Maternal age (years)	31.8 ± 4.9	32.5 ± 5.1	0.38
Nulliparous	224 (40.3)	15 (34.1)	0.64
Previous cesarean scar	229 (41.2)	17 (38.6)	0.74
Uterine malformation	13 (2.3)	1 (2.3)	0.98
BMI before pregnancy (kg/m²)	26.0 ± 6.6	24.2 ± 5.5	0.07
Obesity (BMI, ≥30 kg/m²)	126 (22.9)	5 (11.4)	0.08
Geographic origin			0.45
Caucasian	492 (88.5)	39 (88.6)	
North Africa	12 (2.2)	3 (6.8)	
Sub-Saharan Africa	15 (2.7)	1 (2.3)	
Asian	10 (1.8)	0	
Overseas Departments	8 (1.4)	1 (2.3)	
Hispanic	2 (0.4)	0	
Previous PPH	13 (2.3)	5 (11.4)	<0.001
Twin pregnancy	31 (5.6)	2 (4.5)	0.77
Gestational weight gain (kg)	11.7 ± 6.6	13.3 ± 5.3	0.15
Gestational diabetes mellitus	103 (18.5)	8 (18.2)	0.68
Pregnancy-associated hypertensive disorders	29 (5.2)	3 (6.8)	0.65
Intrahepatic cholestasis of pregnancy	9 (1.6)	0	0.39
Placenta praevia	15 (2.7)	3 (6.8)	0.12
Antenatal suspicion of macrosomia	102 (18.3)	10 (22.7)	0.47
Antenatal polyhydramnios	22 (4.0)	1 (2.3)	0.57
Pre-delivery hemoglobin (g/dL)	11.9 ±1.0	12.1 ± 1.1	0.21
Pre-delivery hematocrit (%)	35.7 ± 3.0	36.0 ± 3.0	0.48

BMI, body mass index. Data are mean ± standard deviation or *n* (%) unless otherwise specified. Student’s t test, χ² test, nonparametric Mann–Whitney test, and Fisher’s exact test were used as appropriate. A *p* value of 0.05 was considered significant.

**Table 2:** Labor characteristics and maternal and neonatal outcomes according to the carbetocin efficacy.

	No Failure (*n* = 556)	Failure (*n* = 44)	*p*-Value
Gestational age at birth (weeks)	38.7 ± 1.8	39.3 ± 2.0	0.07
Neuraxial anesthesia	537 (96.6)	43 (97.7)	0.29
Planned cesarean section	403 (72.5)	24 (54.5)	0.01
Placenta praevia	15 (2.7)	3 (6.8)	
Previous cesarean scar ≥ 2	200 (36.0)	5 (11.4)	
Twin pregnancy	16 (2.9)	2 (4.5)	
Breech presentation	70 (12.6)	4 (9.1)	
Antenatal suspicion of macrosomia	102 (18.3)	10 (22.7)	
Cesarean section during labor	153 (27.5)	20 (45.5)	0.01
Fetal distress	98 (17.6)	12 (27.3)	
Failure to progress	55 (9.9)	8 (18.2)	
Induced labor	169 (30.4)	23 (52.2)	<0.001
Cervical ripening	104 (18.7)	14 (31.8)	0.02
Using a cervical ripening balloon	51 (9.1)	7 (15.9)	0.01
Using a vaginal dinoprostone	53 (9.5)	7 (15.9)	0.04
Induced labor with oxytocin	65 (11.7)	9 (20.5)	0.09
Duration of labor (h)	7.5 ± 4.7	8.2 ± 5.5	0.54
Oxytocin usage during labor	100 (18.0)	11 (25.0)	0.26
Cervical dilatation at the cesarean delivery (cm)	2.4 ± 3.5	4.2 ± 4.1	0.01
Chorioamnionitis	7 (1.3)	0	0.45
Birth weight (g)	3192 ± 609	3451 ± 592	0.01
Delta hemoglobin (g/dL)	1.4 ± 1.0	3.0 ± 1.2	<0.001
Delta hematocrit (%)	3.6 ± 3.0	8.5 ± 3.5	<0.001
Oral iron supplementation	321 (57.7)	34 (77.3)	0.01
Intravenous iron supplementation	8 (1.4)	7 (15.9)	<0.001
Blood transfusion	1 (0.2)	5 (11.4)	<0.001
ICU admission	6 (1.1)	1 (2.3)	0.48
Length of stay in hospital	5.8 ± 2.0	6.0 ± 2.0	0.39

ICU, intensive care unit. Data are mean ± standard deviation or *n* (%) unless otherwise specified. Student’s t test, χ² test, nonparametric Mann–Whitney test, and Fisher’s exact test were used as appropriate. A *p* value of 0.05 was considered significant.

**Table 3:** Multivariate analysis of carbetocin failure after cesarean deliveries.

	Crude OR (CI 95%)	Adjusted OR (CI 95%)	*p*-Value
Previous PPH	5.36 (1.65–15.02)	3.69 (0.50–17.40)	0.20
Cesarean section during labor	2.19 (1.17–4.08)	0.38 (0.07–1.90)	0.20
Cervical dilatation at the cesarean delivery	1.13 (1.04–1.23)	1.22 (1.00–1.50)	0.05
Cervical ripening	2.03 (1.01–3.89)	2.23 (1.01–4.8)	0.04

## Data Availability

The data presented in this study are available on request from the corresponding author. The data are not publicly available due to institutional policies.
